# Effect of the High Hydrostatic Pressure Process on the Microbial and Physicochemical Quality of Shalgam

**DOI:** 10.1021/acsomega.3c08297

**Published:** 2024-02-19

**Authors:** Eylül Ozturk, Hami Alpas, Muhammet Arici

**Affiliations:** † Food Engineering Department, Yildiz Technical University, Istanbul 34220, Turkey; ‡ Food Engineering Department, Middle East Technical University, Ankara 06800, Turkey

## Abstract

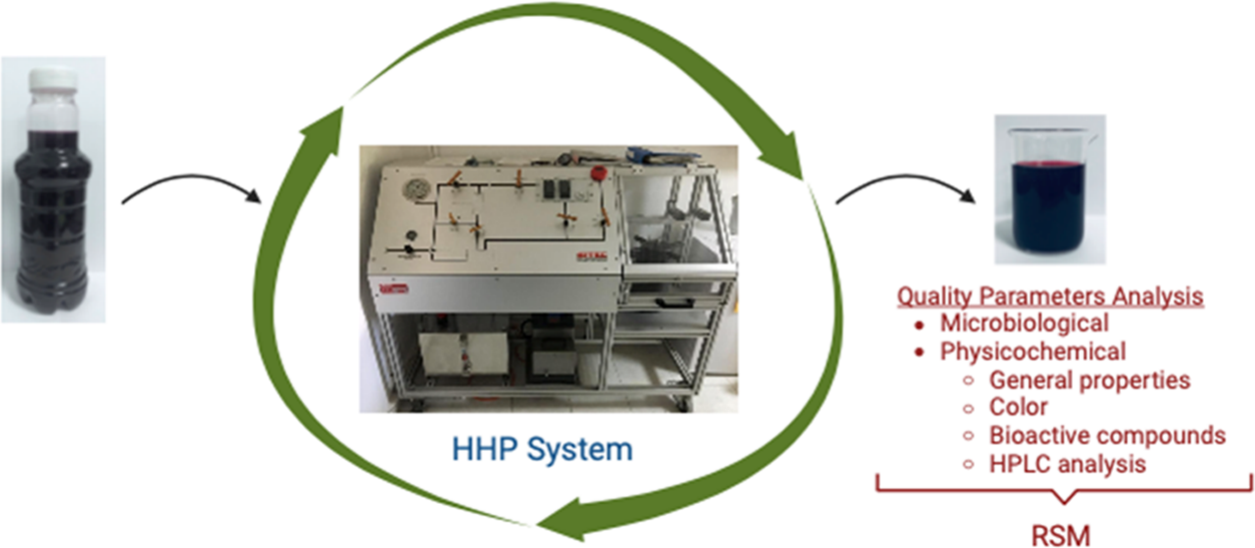

The processing of shalgam requires the use of an appropriate processing technique due to yeast overgrowth. With advancements in processing technology, high hydrostatic pressure (HHP) as nonthermal and nonchemical preservation has gained attention for its potential. Response surface methodology with the Box–Behnken experimental design was used to make sense of the effects of HHP parameters, namely, pressure (100–500 MPa), temperature (20–40 °C), and time (5–15 min), on microbial and physicochemical factors (pH, total soluble solids, titratable acidity, bioactive compounds, color values). The reduction in the counts of total mesophilic aerobic bacteria, lactic acid bacteria, and yeast-mold increased proportionally with the increase of all pressure levels, application temperatures, and pressurization times (*p* < 0.05). Stability was maintained in pH, total solubility, and some color parameters such as L*, a*, Δ*E*, yellow color tone, and red color tone. All findings of the bioactive components (phenolic content, flavonoid content, antioxidant activity, and monomeric anthocyanin content) in the RSM design showed a significant change only in proportion to the square of time (*p* < 0.05). The optimum pressurization parameter combination of shalgam was determined as a pressure of 367 MPa, temperature of 31.9 °C, and process time of 10.5 min. Under these conditions, values of yeast and mold (Y&M) reduction, total flavonoid content (TFC), total monomeric anthocyanin contents (TMACs), titratable acidity (TA), and reducing sugar content (RSC) were obtained as 4.30 log cfu/mL, 192.89 mg QE/100 mL, 11.88 mg/100 mL, 2.41 g_lactic acid_/L, and 6.78 mg/100 mL, respectively. In particular, the findings in the basic color parameters proved that there was no significant change in the saturated red color of the shalgam. Gallic acid, caffeic acid, chlorogenic acid, catechin, cyanidin-3-O-glucoside, malvidin-3-O-glucoside, and peonidin-3-O-glucoside derivatives are dominant phenolic and anthocyanin compounds, which are frequently found in turnip plants. No important losses in bioactive components were observed, despite changes in pressure and temperature parameters. The HHP method can be suggested to have great potential in the processing of shalgam (fermented turnip beverage) in terms of its ability to maintain the flavors, colors, and nutrients, in addition to ensuring microbiological safety when compared to other preservation methods.

## Introduction

Shalgam, a purplish-red-colored and sour beverage, is produced by lactic acid fermentation, mainly from turnip (*Brassica napus* var. napobrassica) and black carrot (*Daucus carota* ssp. *sativus* var. Atrorubens Alef.) originating from Turkey, the Middle East, and the Far East.^1,2^ Shalgam is known especially to be a traditional Turkish beverage (from the Adana region), and its production involves lactic acid fermentation of a mixture of black carrot, turnip, bulgur flour, rock salt, and baker’s yeast. Shalgam gets its intense purplish-red color from black carrots, which contain high amounts of antioxidants.^3,4^ Recent research has highlighted the potential health benefits of consuming shalgam. Composed mainly of cyanidin-based pigments, black carrot has also proven its high antioxidant levels (a large group of red-blue polyphenolic pigments), known for its ability to prevent various diseases, and is used as a natural food coloring due to some of its properties (high temperature, light, and pH stability).^3,4^ Kammerer et al.^1^ claimed that the acylated anthocyanin values in different black carrot samples were determined to be in the range of 55–99% of the total anthocyanin content.

The processing of shalgam, like other juices, requires the use of an appropriate processing technique because yeast overgrowth is a potential risk for unprocessed fermented products in general. Preservation of fermented beverages is a very crucial issue. The sour taste of shalgam is obtained by lactic acid, which inhibits bacterial growth, but it has no effect on preventing yeast growth, and the product should be consumed in a short time. On the other hand, yeast activation can cause problems in long-term uncontrolled storage or transportation conditions.^3^ Therefore, the limited techniques used to protect this fermented product against microbial spoilage are pasteurization and the use of permitted additives (benzoic acid, etc.). It is a known situation that heat treatment (pasteurization) used for commercial purposes causes some undesirable effects on physical, functional, and chemical properties. The biggest reason for this is the high application temperatures. In order to avoid both the use of chemical additives and the undesirable effects of heat treatment and to prevent loss in the quality parameters of shalgam, new, nonthermal, and nonchemical preservation methods should be applied.^3,5^

With advancements in food processing technologies, the use of high hydrostatic pressure (HHP) has gained attention for its potential. High hydrostatic pressure, also known as cold pasteurization or high-pressure processing, involves subjecting food and beverages to high levels of pressure to eliminate microorganisms and extend the product’s shelf life.^6−8^ Studies have demonstrated that high hydrostatic pressure treatment can effectively reduce the microbial load in beverages, including yeasts, molds, and vegetative cells of bacteria that may be present in beverages^9,10^ besides spoilage microorganisms and pathogens such as *Salmonella*, *Listeria monocytogenes*, *E. coli* O157:H7, and *Staphylococcus aureus*.^11−13^ This microbial reduction, essential for ensuring the safety of beverages and preventing foodborne illnesses, is obtained by causing changes in protein dynamics and protein–protein interactions with HHP, in other words, by disrupting proteins without any temperature change.^14−16^ In addition, HHP is known to have important effects, especially on noncovalent bonds, hydrophobic interactions, and hydrogen bonds.^17^

This alternative to thermal processing has shown promising results in terms of maintaining the nutritional, functional, and sensory qualities of beverages. One of the key advantages of HHP treatment is its ability to maintain the flavors, taste, and nutrients of beverages.^18^ In an experimental research by Xu et al.,^19^ HHP-processed Se-enriched kiwifruit juices had no significant differences in the total Se content and also in the chemical-physical qualities of total soluble solids, viscosity, titratable acid, and pH during the storage period. The use of HHP does not result in significant deterioration of flavor, color, and texture since it does not break covalent bonds.^20,21^ Furthermore, this method can also lead to the development of a unique food rheology, contributing to the overall quality and drinking experience. Additionally, high hydrostatic pressure treatment has been found to have a minimal impact on the antioxidant activity and polyphenol content of beverages. In one of the previous studies with pomegranate juice, no significant decreases were observed in the antioxidant activity, total phenolic content, and monomeric anthocyanin pigment concentrations for all pressure levels (200, 300, 400 MPa), while significant declines were observed for thermal treatment.^22^ Torres-Ossandón et al.^23^ claimed in their study the retention of the antioxidant level of grape juices after pressurization over 300 MPa for 2 min, although both ORAC and DPPH values decrease slightly after HHP treatment compared to the control samples. Overall, high hydrostatic pressure treatment is a promising method for improving the safety and shelf life of beverages while maintaining their nutritional and sensory qualities.

A broad range of results belonging to previous studies on shalgam has been obtained regarding microbiological deterioration, color, and bioactive component content using conventional techniques. Researchers found that when the optimum conditions derived from the Box–Behnken design were implemented, a 0.91 log cfu/mL reduction in the total mesophilic aerobic bacteria count (TMAB) and a 0.87 log cfu/mL reduction in the yeast-mold count were attained in a study on shalgam samples processed using the HHP method, which is distinct from the traditional method.^24^ It has been observed that the results obtained in many HHP studies contradict the results of this previous study in this sense. In addition, the fact that the pressure, one of the application parameters of the determined optimum condition (500 MPa, 34.23 °C, 15 min), was at the upper limit of the range value made us think that this study should be planned and the results should be compared.

The aim of this study is both to apply the nonthermal high hydrostatic pressure process, as an alternative to thermal pasteurization and to the usage of additives to prevent microbial spoilage and yeast overgrowth, to shalgam juice and also to determine the effects of this process on microbiological, physicochemical, and bioactive component parameters. HHP application was applied to shalgam juice samples at 20–40 °C for 5–15 min, at 100–500 MPa, with certain combinations of treatments. After the cold pasteurization process, necessary microbiological, physicochemical, bioactive component, and phenolic/antocyanin profile analyses (HPLC analysis) were performed, and the results of the analysis were evaluated statistically by using both the response surface method (RSM) and ANOVA.

## Materials and Methods

### Materials

Shalgam beverages, produced by using a traditional method without using any permitted additives or applying any pasteurization treatment, were procured from the Food Engineering Department, Cukurova University (Adana, Turkey). These samples were provided at proper temperature conditions (below 10 °C), and the series of analyses were processed immediately after the transfer.

### High Hydrostatic Pressure (HHP) Application

In the treatment of shalgam with the HHP treatment, the Box–Behnken experimental design was arranged and applied. While planning the design, pressure, temperature, and time in the ranges of 100–500 MPa, 20–40 °C, and 5–15 min were used, respectively. These parameters were evaluated in light of the results obtained from a previous study and in line with the capacity of the machine used. A laboratory-scale unit (SITEC-Sieber Engineering AG type 760.0118, Zurich, Switzerland) in the Department of Food Engineering at Middle East Technical University was used to conduct HHP experiments. The pressure container, which had a capacity of 100 mL, possessed an internal diameter of 24 mm and a length of 153 mm. It was filled with distilled water to serve as a medium for transmitting pressure. The pressure control mechanism had a rate of increasing pressure at 75 MPa/min up to 100 MPa and at 300 MPa/min up to 500 MPa. Pressure release times were kept below 20 s. To maintain the desired temperature inside the container, a heating–cooling system called the Huber Circulation Thermostat from Offenburg, Germany, was utilized.

### Experimental Design with RSM

Optimization studies were performed using response surface methodology (RSM) as a function of pressure (100–500 MPa), time (5–15 min), and treatment temperature (20–40 °C). This method was figured with a Box–Behnken experimental design with a quadratic model. The HHP process parameters and their Box–Behnken experimental design are given in Table 1. The designation of the optimum condition for pressurization is the main statistical approach to observe both the interaction between different process conditions and the effect of the collective response of different quality parameters.
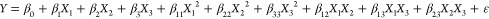
1

**Table 1:** Box–Behnken Experimental Design for HHP Conditions (Natural/Uncoded)/Experimental Results for Reduction Values of TMAB, LAB, and Y&M of Shalgam Samples after HHP Application^a^,^b^

	independent variables			responses		
run	pressure (MPa)	temperature (°C)	time (min)	TMAB (log cfu/mL reduction)	LAB (log cfu/mL reduction)	Y&M^b^ (log cfu/mL reduction)
1	100	20	10	2.46 ± 0.14^g^	0.61 ± 0.21^ghı^	1.02 ± 0.36
2	500	20	10	4.99 ± 0.21^abc^	6.68 ± 0.00^a^	2.86 ± 0.07
3	100	40	10	4.80 ± 0.04^cde^	0.76 ± 0.08^gh^	1.40 ± 0.21
4	500	40	10	5.41 ± 0.14^ab^	6.68 ± 0.00^a^	4.63 ± 0.00
5	100	30	5	1.57 ± 0.21^h^	0.82 ± 0.05^g^	1.05 ± 0.11
6	500	30	5	5.00 ± 0.10^abc^	6.68 ± 0.00^a^	3.24 ± 0.09
7	100	30	15	3.57 ± 0.10^f^	1.64 ± 0.21^f^	1.13 ± 0.10
8	500	30	15	5.46 ± 0.04^a^	6.68 ± 0.00^a^	4.63 ± 0.00
9	300	20	5	2.56 ± 0.34^g^	1.89 ± 0.03^d^	2.80 ± 0.16
10	300	40	5	4.40 ± 0.27^g^	3.43 ± 0.22^c^	3.33 ± 0.00
11	300	20	15	5.19 ± 0.06^abc^	6.68 ± 0.00^a^	3.09 ± 0.06
12	300	40	15	5.22 ± 0.10^abc^	6.68 ± 0.00^a^	4.63 ± 0.00
13	300	30	10	5.11 ± 0.06^abc^	5.58 ± 0.17^b^	3.63 ± 0.00
14	300	30	10	5.11 ± 0.09^abc^	4.98 ± 0.07^b^	3.93 ± 0.04
15	300	30	10	5.22 ± 0.13^abc^	5.20 ± 0.13^b^	3.89 ± 0.07

a Different small letters represent significant differences (*p* < 0.05).

b RSM method was performed.

Minitab 19 software (Minitab Inc., Penn State) was used for graphical and statistical design. A second-order polynomial model (eq 1) was used in order to explain the relationship between the independent and dependent factors. HHP parameters were expressed as coded variables, and the response functions were represented as *Y*. The polynomial model’s regression coefficients are denoted by the letters β_0_ for the constant term, β_*i*_ for linear effects, β_*ii*_ for quadratic effects, and β_*ij*_ for interaction effects. Random error was represented by the term ε. The effectiveness of the model was assessed using analysis of variance (ANOVA), which was also used to calculate the statistical significance of the regression coefficients. To represent the impact of a single process parameter or the interplay of several parameters on each examined response, a mathematical model was developed. Utilizing the same software, a 3D surface plot was created using the function of two parameters while holding the others constant.

### Microbiological Analysis

The determination of inactivation of the natural microflora of shalgam samples was performed by total mesophilic aerobic bacteria, total yeast-mold, total lactic acid bacteria, and total Enterobactericeae counts. Before all treatments, shalgam samples were diluted (1:10 diluent, physiological saline, NaCl solution 0.85%) to determine microbial counts and inoculated into the respective medium for each microorganism. For total mesophilic aerobic bacteria count (TMAB), diluted samples were inoculated on plate count agar (PCA) and incubated on these plates at 35 ± 2 °C for 24–48 h. For total yeast and mold counts (Y&M), potato dextrose agar (PDA) was prepared and inoculated on agar and obtained after incubation at 22 ± 2 °C for 2–5 days for determination. For lactic acid bacteria (LAB), Man Rogosa Sharpe (MRS) medium (Merck, Darmstadt, Germany) was prepared and inoculated from appropriate dilutions. Count results were obtained after 3–5 days of incubation at 30 ± 2 °C. For the determination of total Enterobacteriaceae (TE) count, violet red bile (VRB) agar (Merck, Darmstadt, Germany) was prepared. After the inoculations, the Petri dishes were incubated at 35 ± 2 °C for 24–48 h and the results were obtained. All results were obtained by performing three parallel inoculations.

### Physicochemical Properties

#### pH, Total Soluble Solids, and Titratable Acidity Analysis

pH and total soluble solid values of shalgam samples were measured at room temperature using a pH meter (WTW pH7110, Xylem Analytics, Germany) and digital refractometer (HANNA Instruments HI96801), respectively. The titratable acidity (TA) (lactic acid equivalent) was determined according to the general procedure of the titrimetric method.^25^

#### Color Analysis

Color analysis of the samples was performed using a Konica Minolta (Hunter Associates Laboratory Inc. Reston VA) device. Color values were stated as L* (brightness and darkness), a* (redness and greenness), and b* (yellowness and blueness) values. Other color values, total color difference (Δ*E*), color intensity (C*, chroma), and hue (*h*°) were derived with equations including the L*, a*, and b* values.

Moreover, the color intensity (IC), the color tone (CT), and the percentage of color components (yellow, blue, and red) were measured with the absorbance values (420, 520, and 620 nm) of the remaining supernatant of shalgam samples, which was centrifuged at 5000 rpm for 5 min (OPTIZEN POP UV/vis spectrophotometer, Mecasys Co. Ltd., Korea). These results were obtained at different specific wavelengths in terms of colors, yellow (YCT, OD_420_), red (RCT, OD_520_), and blue (BCT, OD_620_) color tones, against the reference sample (distilled water).^26^ Color intensity (IC = OD_420_ + OD_520_ + OD_620_) and color tone (CT = OD_420_/OD_520_) were calculated by using the color components measured.

#### Reducing Sugar Analysis

The DNS (dinitrosalicylic acid) method, introduced by Miller in 1959, was utilized for determining the reducing sugar content (RSC). In this procedure, DNS reagents were mixed with the sample solutions in a 1:1.5 volume-to-volume ratio. The mixture was then placed in a water bath (the Witeg Baths WCB Circulation Water Bath, Wertheim, Germany) and subjected to temperatures of 90–100 °C for a duration of 5–8 min. Once the desired color change was observed (indicating the transformation of the yellow color to orange or dark orange), the samples were transferred to an ice bath for 5 min. Finally, the absorbance of the samples was measured by using a UV-spectrophotometer at a wavelength of 575 nm.

#### Bioactive Compound Analysis

Total phenolic content (TPC) analysis in the studies was carried out according to the Folin–Ciocalteu method, which is a common method. The diluted shalgam samples (0.5 mL) were mixed with Folin–Ciocalteu’s phenol reagent (1:10 v/v) and Na_2_CO_3_ (7.5 g/100 g) in quantities of 2.5 and 2 mL, respectively. The mixtures were in the dark for 30 min, and so an absorbance reading at 760 nm was obtained using a UV–vis spectrophotometer (specifically, the Shimadzu UV-1800 model from Kyoto, Japan). The phenolic content was calculated in gallic acid equivalents per liter of shalgam samples (mg GAE/L).

The total flavonoid content (TFC) was evaluated with a modified method based on the procedure described by Zhishen et al.^27^ A 0.03 mL portion of 5% NaNO_2_ solution was combined with 0.4 mL of appropriately diluted shalgam samples. After a 5 min incubation, 0.3 mL of 1% AlCl_3_ was added to the mixture and allowed to incubate again at room temperature for 6 min. Subsequently, 0.2 mL of 1 M NaOH and 0.07 mL of distilled water were added. The absorbance values of the mixtures were read at 510 nm using a spectrophotometer, and the total flavonoid content was given as milligrams of quercetin equivalents (QE) per 1 mL of shalgam. All samples were analyzed in triplicate for all analyses.

For evaluation of the total antioxidant capacity/activity (TAA) of the extracts, the copper-reducing antioxidant capacity (CUPRAC) method was employed.^28^ For the test, a mixture of 1 mL of CuCl_2_ (10 mmol/L), 1 mL of neocuproine (7.5 mmol/L), and 1 mL of NH_4_Ac (1 mol/L) with 0.1 mL of diluted sample was prepared. Subsequently, 1 mL of distilled water was added to this solution to make a total volume of 4.1 mL. The absorbance values were then measured at 450 nm after an incubation period of approximately 1 h in the dark. The results were calculated in trolox equivalents per milliliter of sample (μmol TE/mL).

The total monomeric anthocyanin contents (TMACs) of shalgam samples were determined according to the pH-differential method developed by Giusti and Wrolstad.^29^ 0.1 mL was taken from the samples and added to glass test tubes; to this, 2.4 mL of a 0.025 M KCl solution, adjusted to pH 1.0 with HCl, or a 0.4 M C_2_H_3_NaO_2_ solution, adjusted to pH 4.5 with HCl, was mixed. Both pH 1.0 and 4.5 samples were kept in the dark for 15 min after mixing in a vortex. The absorbance measurements were made after the spectrophotometer was taken as a reference with KCl and C_2_H_3_NaO_2_ solutions. The absorbance was measured at 520 nm, the wavelength at which black carrot anthocyanins show maximum absorbance, and at 700 nm to determine turbidity using a UV-spectrophotometer. The results were expressed as milligrams of cyanidin-3-glucoside per liter (mg of cyanidin-3-glucoside/L) of the sample.

#### Phenolic Acid and Anthocyanin Profile Analysis

The shalgam samples were diluted at an appropriate rate with methanol overnight and then filtered through a 0.22 μm filter. Chromatographic analyses were performed on high-performance liquid chromatography equipment (Shimadzu, Kyoto, Japan), consisting of a photodiode array detector, a quaternary pump, an autosampler, and a column oven, with a Waters Atlantis C18 column (250 × 4.6 mm, 5 μm). Phenolic acids (gallic acid, caffeic acid, p-coumaric acid, ferulic acid, chlorogenic acid, and catechin) were separated with a column by using a linear gradient elution program with a mobile phase containing solvent A (acetic acid/H_2_O, 0.1:99.9, v/v) and solvent B (acetic acid/acetonitrile, 0.1:99.9, v/v) at a flow rate of 1 mL/min. The chromatograms were recorded at 278, 320, and 360 nm. Anthocyanins (delphinidin-3-O-galactoside, cyanidin-3-O-glucoside, malvidin-3-O-glucoside, and peonidin-3-O-glucoside) were used for anthocyanin separation and quantification with a column by using a linear gradient elution program with a mobile phase containing solvent A (formic acid/acetonitrile, 7.5:92.5, v/v) and solvent B (formic acid/H_2_O, 7.5:92.5, v/v) at a flow rate of 1 mL/min; the chromatograms were recorded at 520 nm. Contents and quantities of phenolic acids and anthocyanins were determined according to the retention time and absorption spectra of peaks in samples compared to those of standard compounds and their calibration curves.

### Statistical Analysis

All microbiological and physicochemical responses were analyzed statistically according to independent variables (pressure, temperature and time values) as the functions of linear, quadratic, and also interaction terms by using the Box–Behnken experimental design (Tables 1–3). The evaluation was conducted to take into consideration analysis of variance of the model in terms of *R*^2^ values (coefficient of determination) of variables, lack-of-fit value of the model, and p-values. For the response with a significant lack-of-fit value in the RSM model, ANOVA was carried out to determine the similarity or differences between the samples in the Box–Behnken design. Minitab 19 (Minitab Inc., Penn State) was used for statistical analysis.

## Results and Discussion

### Effect of HHP on Microbial Inactivation

The mean TMAB, LAB, and Y&M count results were determined as 8.06, 6.68, and 4.63 log cfu/mL, respectively, in the microbiological analyses performed on the control sample representing the natural microflora of shalgam. In the TE count, no colonies were observed in any sample before and after the treatment (control sample). The reductions of the population of TMAB, LAB, and Y&M in shalgam samples after HHP treatment at different combinations of pressure level, temperature, and treatment time are shown in Table 1.

The inactivation intensity of TMAB was varied in direct relation with all HHP parameters according to the ANOVA results (*p* < 0.05). The increase of the pressure level, treatment temperature, and time including linear and interaction of some parameters improved the reduction of TMAB. Similarly, Mert et al.^6^ emphasized that the inactivation efficiency was enhanced with the rise in pressure level from 150 to 250 MPa at 30 °C for 5 min. Ates et al.^24^ mentioned in their study on shalgam that 0.91 and 0.74 log cfu/mL reductions in the TMAB count were obtained with optimum HHP application conditions for spicy (500 MPa, 34.23 °C, 15 min) and sweet (363.6 MPa, 40 °C, 15 min) tastes. When comparing the previous results of sweet shalgam juice, it was observed that our findings show a more effective inactivation (>5.22 log reduction). The Food Safety and Inspection Service (FSIS) in the USA mandates a 5-log reduction for L. monocytogenes, a high-mortality pathogen, in ready-to-eat meat products after treatment.^30^ Even if the total inactivation could not be achieved due to the applied parameter ranges, 5.41 log cfu/mL reduction was detected in the case of HHP application at 500 MPa, 40 °C for 10 min, and it was concluded that this reduction that was obtained by using a treatment method was at the expected level. In another study with pomegranate juice, total inactivation could be obtained with 3.85 log cfu/mL reduction for TMAB at pressurization conditions as 300 MPa to 10 min, 5 min, 15 °C, and 400 MPa to 5 min, 5 min, 15 min, 25 °C.^31^

In the same way, the inactivation of LAB was dependent on the level of HHP parameters in consideration of the ANOVA results. The results demonstrated the effect of HHP was significantly promoted with rises in pressure level and application time (*p* < 0.05). Total inactivation (6.68 log cfu/mL) was obtained in combinations of 300 MPa for 15 min and higher levels, including all temperature values. While a decrease of more than 1 log cfu/mL was obtained for LAB in wine samples with the application of 400 and 500 MPa for 15 min,^32^ inactivation quantities were obtained of more than 4.0 log cfu/mL reduction in tomato juice with 500 MPa-3 min application^33^ and 6.51 log cfu/mL reduction in aloe vera juice in the HHP at 400–600 MPa for 10–30 min,^34^ considering that it varies depending on the product. Moreover, in the study performed on shalgam, 1.71/1.59 log cfu/mL and 1.28 and 1.40 log cfu/mL reductions were found for *Lactococcus lactis* subsp. *cremoris* and *Lactobacillus paracasei* at 500 MPa–34.23 °C–15 min/363.6 MPa–40 °C–15 min conditions. Parallel to our observation, the inactivation effect of HHP indicated an increasing trend only for application time and pressure significantly.^24^

**Figure 1:**
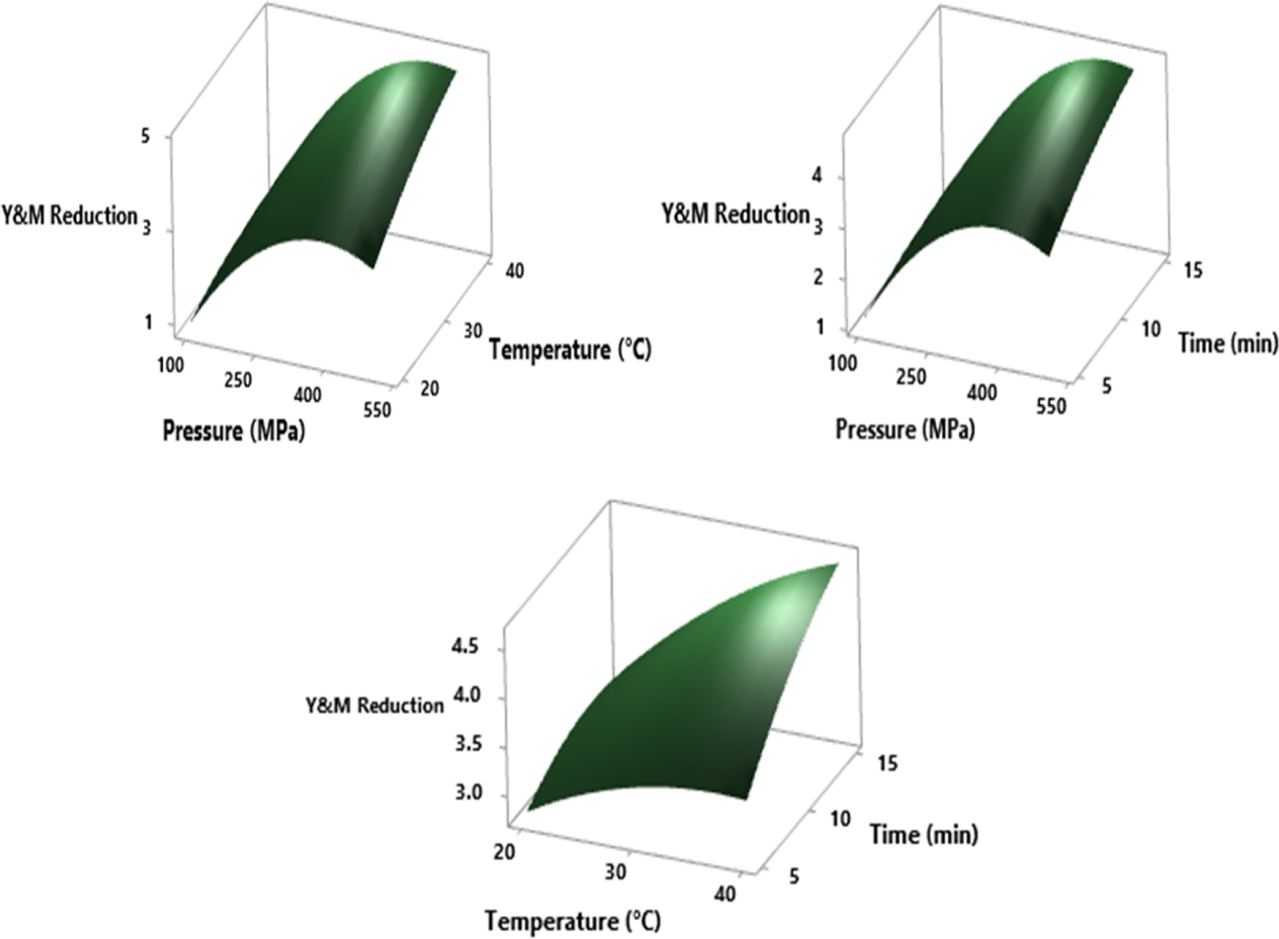
RSM plots of the HHP effect on Y&M reduction in cross-interactions among pressure, temperature, and time parameters at hold values (pressure 300 MPa, temperature 30 °C, time 10 min).

When the results of the detection of yeasts, as the most important microflora element to focus on for shalgam, were evaluated according to the ANOVA analysis of the variance table of Y&M counts, it was seen that both the linear and second-order powers and their interactions of the pressure level, application time, and temperature parameters were effective on microorganism reduction (*p* < 0.05). The increase in all parameters provides a significant increase in the inactivation efficiency, and in addition, it has been seen that the interactions of pressure and other parameters have a more affirmative effect than the time–temperature interaction (*p* = 0.005, *p* = 0.006, < *p* = 0.017). Figure 1 shows the reductions of Y&M counts with pressure–temperature, pressure–time, and temperature–time at a fixed time of 10 min, temperature 30 °C, and pressure 300 MPa, respectively. Under the experimental circumstances of pressure ∼350 to 450 MPa, temperature ∼30 to 40 °C, and duration ∼10 to 15 min, the graph plot showed at maximum grade that the Y&M reduction increased (Figure 1).

In the study of Chang et al.^35^ with grape juice, a 1.1 log cfu/mL reduction was detected in a 300 MPa 3 min application, and the total inactivation level was reached when the applied pressure was raised to 600 MPa (>2.0 log). In another previous study, when pressure levels of 400 MPa and above were applied to raspberry juice for 2, 5, and 10 min, total inactivation was obtained with a 3.19 log cfu/mL reduction.^36^ According to a study on shalgam, the number of total yeast and molds was seen to linearly increase with rising temperature, regardless of the application pressure.^24^ An increase in the number of total yeast and molds was found as the application time was extended from 3 to 9 min; however, beyond that range, from 9 to 15 min, the number of Y&M is anticipated to decrease with an increase in application time.

### Effect of HHP on pH, Total Soluble Solids, and Titratable Acidity

HHP, which is one of the nonthermal heat treatments, is an alternative application to prevent undesirable quality changes, as has been stated and proven in many studies. In addition to its success in establishing food safety microbiologically, it has been argued that the most important advantage of its application is that it does not cause significant losses in the physicochemical and organoleptical properties of fruit juices.^6,10^ In different previous studies, pH and TSS values were found to have no notable changes just after HHP for various samples such as papaya juice at 350–650 MPa and 5–10 min;^37^ watermelon juice at 200–600 MPa, 5–60 min, and 20 °C;^8^ citrus-maqui beverages at 450–600 MPa, 3 min, and 20 °C;^38^ and white grape juice at 200–400 MPa, 2–4 min, and 20 °C.^23^ In addition to the effects immediately after the process, it has been proven in different products analyzed in many studies that the preservation of these parameters continues throughout the storage period. pH and TSS parameters could keep stable in studies with fermented pomegranate beverage at 550 MPa-10 min and 600 MPa-5 min during 42 days,^10^ with maoberry juice at 400 MPa-10 min-25 °C during 4 weeks,^39^ and with kiwifruit juice at 500 MPa-10 min-25 °C during storage time.^19^ In line with the results of the studies carried out, pH (3.92 ± 0.01–4.17 ± 0.03) and TSS (1.13 ± 0.06–3.50 ± 0.00°Brix) values of our pressurized shalgam samples (Table 2) did not indicate any significant changes under different HHP conditions when analysis of variance tables of RSM were examined (*p* > 0.05).

**Table 2:** Experimental Results for pH, TSS, TA, RSC, and Bioactive Compounds of Shalgam Samples after HHP Application^a^,^b^

	responses							
process number	pH	TSS (°Briks)	TA^b^ (g_lactic acid_/L)	RSC^b^ (mg/100 mL)	TPC (mg GAE/100 mL)	TFC^b^ (mg QE/100 mL)	TAA (μmol TE/100 mL)	TMAC^b^ (mg/100 mL)
C	3.96 ± 0.01^g^	1.83 ± 0.15^ef^	2.40 ± 0.21	5.90 ± 0.09	43.22 ± 0.40^cd^	186.85 ± 23.49	787.67 ± 8.12^a^	13.27 ± 0.57
1	3.92 ± 0.01^h^	3.13 ± 0.06^b^	4.08 ± 0.21	13.47 ± 0.21	34.16 ± 0.42^f^	151.44 ± 2.17	754.72 ± 1.25^bc^	7.45 ± 0.38
2	4.02 ± 0.01^f^	1.73 ± 0.06^fg^	3.12 ± 0.21	8.17 ± 0.07	30.29 ± 0.51^gh^	131.23 ± 7.51	611.21 ± 3.14^h^	7.79 ± 0.64
3	4.05 ± 0.01^ef^	1.90 ± 0.00^e^	3.36 ± 0.21	15.12 ± 0.03	45.04 ± 1.60^bc^	178.73 ± 7.19	763.53 ± 13.75^ab^	11.79 ± 1.24
4	4.08 ± 0.02^cde^	1.90 ± 0.00^e^	2.76 ± 0.21	9.26 ± 0.06	38.69 ± 0.65^e^	161.02 ± 2.19	691.11 ± 5.20^ef^	10.00 ± 0.15
5	4.08 ± 0.03^de^	1.90 ± 0.00^e^	4.44 ± 0.21	14.42 ± 0.16	42.61 ± 0.99^d^	178.73 ± 9.69	749.80 ± 3.14^bc^	11.98 ± 0.81
6	4.11 ± 0.00^bcd^	1.80 ± 0.00^efg^	2.52 ± 0.00	8.45 ± 0.10	29.31 ± 0.88^h^	119.77 ± 14.39	591.23 ± 4.73^hı^	6.63 ± 0.46
7	4.08 ± 0.01^cde^	2.90 ± 0.00^c^	3.24 ± 0.00	11.68 ± 0.02	24.72 ± 0.58^j^	112.90 ± 15.28	643.37 ± 2.16^g^	6.21 ± 0.53
8	4.12 ± 0.00^bc^	1.13 ± 0.06^h^	3.12 ± 0.21	8.84 ± 0.20	28.43 ± 0.55^hı^	129.15 ± 6.86	586.65 ± 10.09^ıj^	6.65 ± 0.14
9	4.07 ± 0.00^e^	3.20 ± 0.00^b^	3.24 ± 0.00	13.41 ± 0.03	26.67 ± 0.40^ıj^	95.60 ± 9.81	455.55 ± 19.40^l^	4.96 ± 0.18
10	4.05 ± 0.02^ef^	3.50 ± 0.00^a^	3.12 ± 0.21	14.65 ± 0.11	31.63 ± 0.91^g^	118.31 ± 7.58	503.83 ± 0.72^k^	6.91 ± 0.13
11	4.17 ± 0.03^a^	1.23 ± 0.06^h^	2.92 ± 0.21	8.41 ± 0.09	37.73 ± 0.24^e^	164.56 ± 7.37	667.39 ± 3.14^fg^	7.83 ± 0.76
12	4.08 ± 0.01^cde^	2.97 ± 0.06^c^	3.24 ± 0.00	12.57 ± 0.08	25.09 ± 0.73^j^	122.69 ± 8.17	562.51 ± 6.28^j^	7.36 ± 0.18
13	4.11 ± 0.00^bcd^	2.10 ± 0.00^d^	2.56 ± 0.21	7.37 ± 0.11	46.00 ± 0.24^ab^	195.01 ± 1.48	738.35 ± 7.49^c^	12.00 ± 0.24
14	4.13 ± 0.00^b^	1.87 ± 0.06^ef^	2.40 ± 0.21	6.78 ± 0.17	47.33 ± 0.86^a^	192.27 ± 1.80	733.57 ± 5.63^cd^	12.96 ± 0.95
15	4.14 ± 0.00^ab^	1.67 ± 0.06g	2.52 ± 0.00	7.48 ± 0.03	45.57 ± 0.44^ab^	206.65 ± 7.46	710.68 ± 8.65^de^	11.60 ± 0.96

a Different small letters represent significant differences (*p* < 0.05).

b RSM was performed.

**Figure 2:**
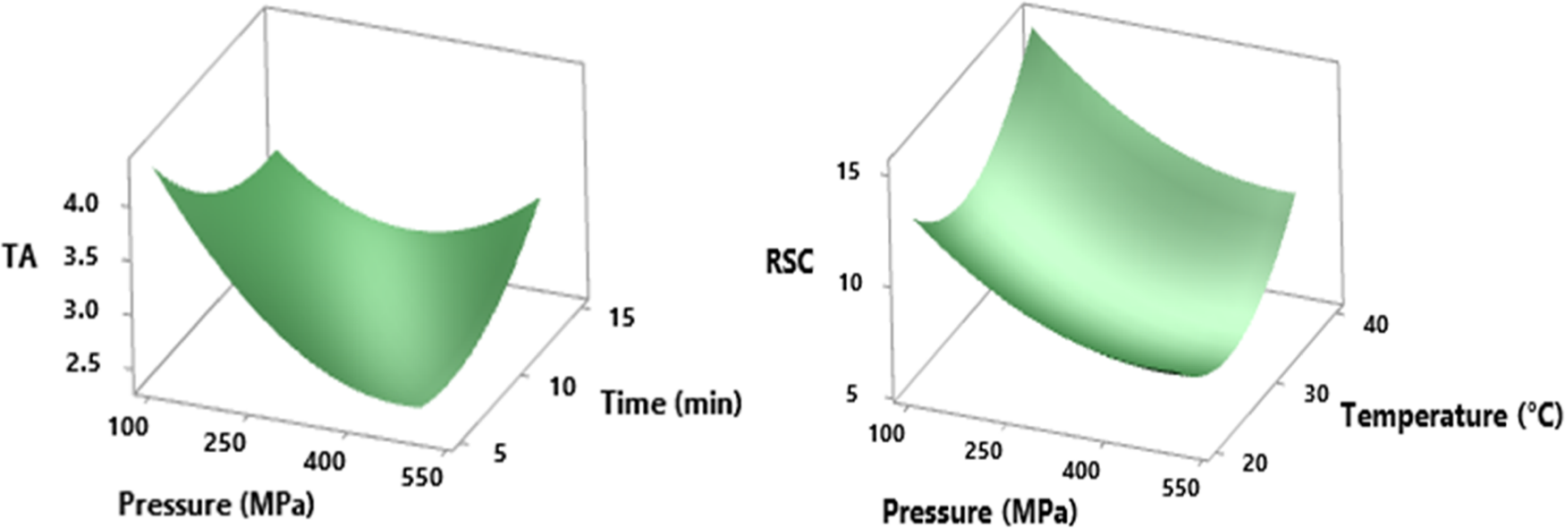
RSM plot of the HHP effect on TA (g_lactic acid_/L) and RSC (mg/100 mL) values during cross-interaction between pressure and time parameters at hold values (temperature 30 °C and time 10 min).

Titratable acidity measurements of untreated shalgam were determined as 2.40 ± 0.21 g/L in terms of lactic acid, considering that it is a lactic acid fermentation product.^40^ Important changes in TA values were observed after pressure treatment, and these changes were related to the pressure level linear, square power, and interaction with application time (*p* < 0.05). The result of a raising effect of interaction, which is inversely proportional to the increase in pressure from 100 to 500 MPa and directly proportional to the increase in application time from 5 to 15 min, can be clearly seen in the 3D surface plot in Figure 2. Liu et al.^41^ emphasized in their experimental results that the TA of strawberry juices at 400 MPa and beyond did not change, but this value increased with an increase in application time at 300 MPa and below. However, the stability of TA values of white grape juice,^35^ cloudy pomegranate juice,^42^ apple juice,^43^ and kiwifruit juice^19^ was provided in comparison with control samples after HHP treatment at 600 MPa-20 °C-3 min, 300–400 MPa-2.5–25 min, 200–400 MPa-10 min, and 500 MPa-10 min-25 °C, respectively. In another study, slight decreases in the range of 0.1–0.26% were observed in the titration acidity values in sugarcane juice samples at higher pressures in the range of microfluidization (150–200 MPa-1 and 3 cycles), while the pH value did not change in the range of 5.2–5.7 when compared to the control sample.^44^

All HHP parameters, such as linear and square power, are significantly effective on the RSC of shalgam and were determined with RSM results. Under the experimental circumstances of pressure ∼300 to 500 MPa, temperature ∼25 to 30 °C, and duration 10–12 min, the 3D graph plot (Figure 2) indicates a minimum increase in the reducing sugar values. Except for these ranges of parameters, the reducing sugar levels show a more increasing tendency with an increment of temperature up to 40 °C and decreases of both pressure from 300 to 100 MPa and treatment time toward 5 min. An increase was observed in the amount of reducing sugar after HHP application, similar to the experimental results we obtained in one of our previous studies on Chinese rice wine.^45^ Although it is known that the pressurization process has both enhancing and stabilizing properties on enzyme activity, it has been stated that the increase in the activation of saccharifying enzymes such as β-glycosidase, β-galactosidase, and α-arabinosidase may be responsible for the increase in the amount of reducing sugar.^15,46^ This enzyme group breaks down anthocyanins into anthocyanidins and sugars and can cause an increase in the amount of reducing sugar.^46^ Ferreira et al.^47^ claimed in their study on opuntia ficus-indica juice that the RSC of HHP-treated juices showed an increasing trend during storage at 4 °C due to the enhanced enzymatic activity on the hydrolysis of polysaccharides and conversion into simple sugars.

### Effect of HHP on Color Parameters

**Figure 3:**
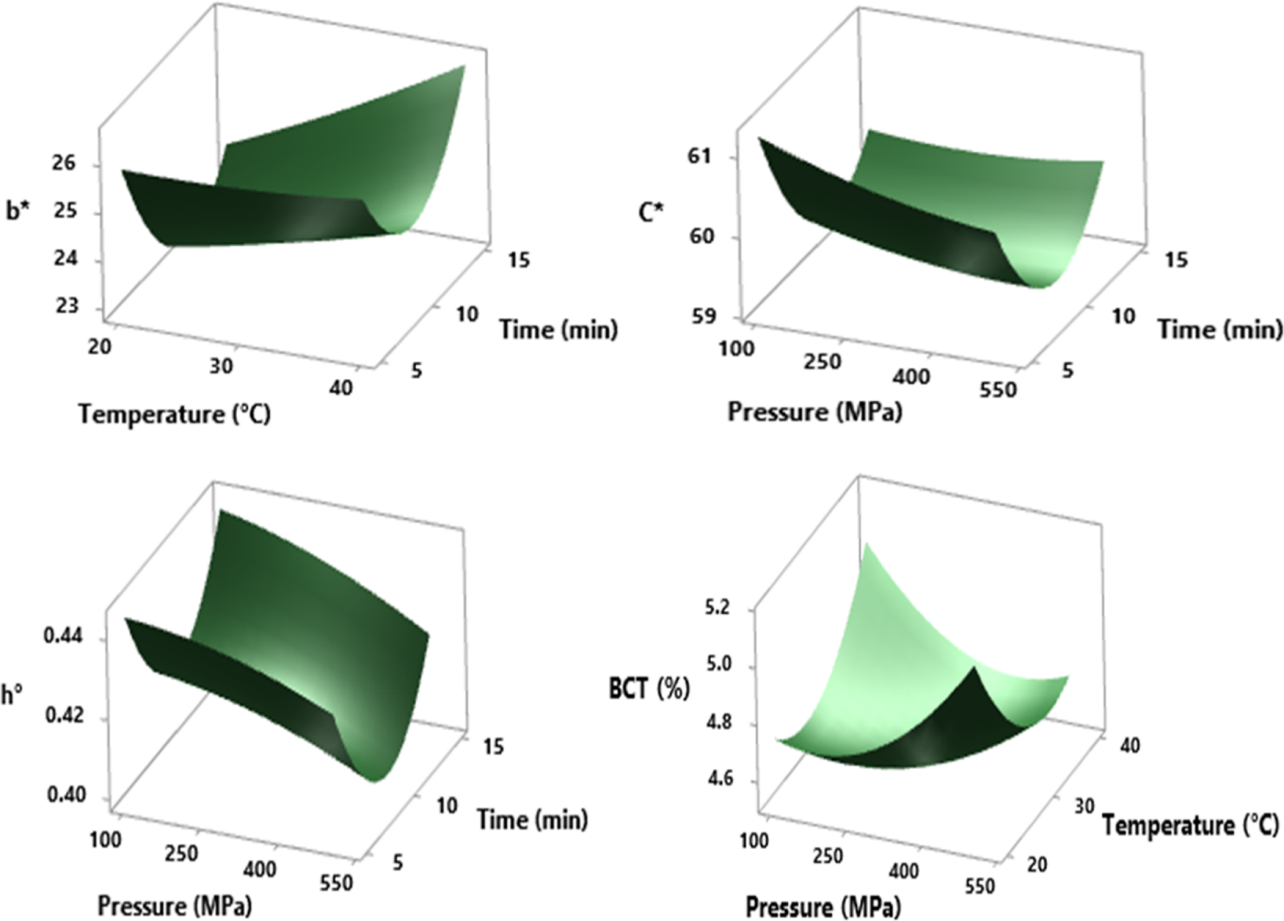
RSM plot of the HHP effect on b*, C*, *h*°, and BCT (%) values in cross-interaction between temperature and time parameters at hold values (pressure 300 MPa, temperature 30 °C, and time 10 min).

All color analysis results are indicated in Table 3. HHP, which has no or minimal effect on most of the quality parameters, also showed the same trend on color quality characteristics, meeting expectations. When the RSM results were evaluated, no significant change was observed in the L* (39.21 ± 1.71–42.92 ± 1.27) and a* (53.39 ± 1.28–55.47 ± 0.81) values of the basic color parameters in terms of the three parameters used in the pressurization process (*p* > 0.05), while a significant change was observed in the b* (21.98 ± 0.47–26.77 ± 0.80) values with the square of time (*p* < 0.05) (Figure 3). In some studies specifically focused on different products such as apple juice,^48^ blended pumpkin–mango–jujube juice,^49^ orange juice,^50^ and lemongrass–lime mixed beverages,^51^ the effect of pressurization was not observed on these three color parameters. However, the highest a* and b* values were obtained just after HHP treatment at 600 MPa for 5 min (24.51 and 11.23) for fermented pomegranate beverage, followed by 550 MPa-10 min (23.99 and 10.56) and 500 MPa-10 min (23.97 and 10.49).^10^ In addition, decreases in the a* and b* values of strawberry juice were seen after pressurization at 400 MPa, but these values also increased relative to treatment time, regardless of the pressure level.^41^

**Table 3:** Experimental Results for Color Parameters of Shalgam Samples after HHP Application^a^,^b^

	responses										
process number	L*	a*	b*^b^	Δ*E*	C*^b^	*h*°^b^	IC	CT	YCT (%)	RCT (%)	BCT (%)^b^
C	39.48 ± 2.29^a^	53.39 ± 1.28^a^	24.30 ± 0.30		58.66 ± 1.20	0.43 ± 0.01	10.63 ± 0.03^fgh^	0.357 ± 0.002^ab^	25.07 ± 0.08^b^	70.06 ± 0.15^bc^	4.87 ± 0.10
1	40.65 ± 1.38^a^	54.32 ± 0.80^a^	24.10 ± 0.50	4.41 ± 1.59^a^	59.43 ± 0.77	0.42 ± 0.01	18.11 ± 0.25^b^	0.345 ± 0.005^bcd^	24.45 ± 0.18^bcd^	70.80 ± 0.46^abc^	4.75 ± 0.27
2	40,46 ± 2.54^a^	54.30 ± 0.84^a^	22.00 ± 5.03	6.59 ± 1.48^a^	58.69 ± 2.56	0.38 ± 0.08	10.60 ± 0.21^fgh^	0.355 ± 0.007^abc^	24.85 ± 0.25^bc^	69.94 ± 0.66^bc^	5.21 ± 0.43
3	42.38 ± 4.56^a^	54.13 ± 2.86^a^	24.21 ± 0.33	6.42 ± 4.39^a^	59.30 ± 2.70	0.42 ± 0.02	10.85 ± 0.09^fg^	0.348 ± 0.002^bcd^	24.53 ± 0.11^bcd^	70.52 ± 0.48^abc^	4.94 ± 0.56
4	43.80 ± 0.31^a^	55.32 ± 0.01^a^	24.39 ± 0.45	7.64 ± 0.28^a^	60.46 ± 0.18	0.42 ± 0.01	10.33 ± 0.09^ghı^	0.345 ± 0.003^bcd^	24.46 ± 0.11^bcd^	70.87 ± 0.28^abc^	4.67 ± 0.17
5	42.92 ± 1.27^a^	55.28 ± 0.35^a^	25.77 ± 0.92	7.04 ± 1.07^a^	61.00 ± 0.07	0.44 ± 0.02	10.50 ± 0.07^fgh^	0.344 ± 0.001^bcd^	24.42 ± 0.06^bcd^	70.98 ± 0.16^abc^	4.60 ± 0.15
6	42.31 ± 3.27^a^	54.44 ± 0.77^a^	24.59 ± 1.67	6.26 ± 2.80^a^	59.75 ± 0.13	0.42 ± 0.03	10.12 ± 0.05^hı^	0.342 ± 0.004^bcd^	24.30 ± 0.20^bcd^	71.16 ± 0.35^ab^	4.54 ± 0.15
7	39.21 ± 1.71^a^	54.67 ± 1.14^a^	26.35 ± 0.76	4.09 ± 2.01^a^	60.69 ± 1.34	0.45 ± 0.00	16.51 ± 0.17^d^	0.341 ± 0.002^bcd^	24.27 ± 0.06^bcd^	71.23 ± 0.17^ab^	4.49 ± 0.11
8	41.43 ± 1.28^a^	54.76 ± 0.24^a^	25.12 ± 0.92	5.41 ± 1.12^a^	60.25 ± 0.18	0.43 ± 0.02	12.61 ± 0.17^e^	0.343 ± 0.002^bcd^	24.37 ± 0.06^bcd^	71.12 ± 0.32^ab^	4.51 ± 0.26
9	40.71 ± 2.89^a^	55.47 ± 0.81^a^	26.39 ± 1.11	5.95 ± 1.62^a^	61.44 ± 0.34	0.44 ± 0.02	18.39 ± 0.22^b^	0.341 ± 0.006^bcd^	24.27 ± 0.24^bcd^	71.15 ± 0.57^ab^	4.58 ± 0.33
10	39.32 ± 2.93^a^	55.21 ± 0.98^a^	26.77 ± 0.80	5.12 ± 1.87^a^	61.37 ± 0.66	0.45 ± 0.02	19.73 ± 0.19^a^	0.338 ± 0.000^cd^	24.08 ± 0.09^cd^	71.25 ± 0.27^ab^	4.67 ± 0.36
11	42.54 ± 0.97^a^	53.47 ± 0.23^a^	23.38 ± 0.41	5.89 ± 1.03^a^	58.36 ± 0.21	0.41 ± 0.01	10.51 ± 0.14^fgh^	0.344 ± 0.006^bcd^	24.42 ± 0.24^bcd^	71.01 ± 0.63^abc^	4.56 ± 0.41
12	39.87 ± 2.81^a^	54.84 ± 0.88^a^	26.05 ± 0.98	4.81 ± 2.05^a^	60.62 ± 0.44	0.44 ± 0.02	17.43 ± 0.45^c^	0.348 ± 0.014^bcd^	24.65 ± 0.73^bcd^	70.88 ± 0.82^abc^	4.47 ± 0.12
13	40.66 ± 3.43^a^	54.07 ± 0.82^a^	25.06 ± 1.51	4.86 ± 2.69^a^	59.61 ± 0.48	0.43 ± 0.03	12.55 ± 0.11^e^	0.341 ± 0.003^bcd^	24.23 ± 0.12^cd^	71.09 ± 0.38^ab^	4.68 ± 0.28
14	42.84 ± 2.90^a^	54.51 ± 0.46^a^	23.98 ± 2.05	6.73 ± 2.66^a^	59.57 ± 0.49	0.41 ± 0.03	10.95 ± 0.09^f^	0.335 ± 0.001^d^	23.99 ± 0.11^d^	71.53 ± 0.14^a^	4.47 ± 0.25
15	44.41 ± 0.22^a^	54.14 ± 0.22^a^	21.98 ± 0.47	8.15 ± 0.30^a^	58.44 ± 0.33	0.39 ± 0.01	9.99 ± 0.16^ı^	0.373 ± 0.013^a^	25.93 ± 0.62^a^	69.63 ± 0.75^c^	4.43 ± 0.16

a Different small letters represent significant differences (*p* < 0.05).

b RSM was performed.

In terms of examination of other color parameters, no important difference in Δ*E* values was found between various conditions (*p* > 0.05). Quiroz-González et al.^52^ and Xu et al.^19^ similarly emphasized that Δ*E* values were stable right after HHP treatment with conditions at 550, 600 MPa for 16, 12 min and 500 MPa-10 min-25 °C for products like pitaya and kiwifruit juice, respectively. However, C* and *h*° values of treated shalgam juices were influenced significantly by changing the square of application time just as mentioned above for b* values (*p* < 0.05). When the C*, *h*°, and b* values are interpreted into the 3D RSM plot (Figure 3), they tend to be very similar in general structure. When evaluated in the model, these color parameters reach their minimum points with the decrease of pressure level and temperature value (approximately 500 MPa-20 °C-10 min). The *h* and *b* values exhibited the same graphic pattern for the maximum points, although slight differences were observed for the maximum value for parameter C*.

When the color intensity (IC), color tone (CT), YCT, RCT, and BCT values were evaluated statistically, no significant changes were observed in the other mentioned parameters (*p* > 0.05) except for the BCT value (*p* < 0.05). This change in BCT values between 4.43 ± 0.16 and 5.21 ± 0.43 was a result of the significant change in blue/yellow values after HHP treatment. Table 4 shows that the square of all HHP parameters and the pressure–temperature interaction are effective on these changes and the minimum BCT value was observed nearly at mid-conditions (200–400 MPa at 25–35 °C for 7–12 min), as shown in the 3D RSM plot in Figure 3. The highest value was recorded at 500 MPa, 20 °C, and 10 min, and the second highest value occurred in conditions that had opposite conditions of the first value in terms of pressure and temperature. Atmaca et al.^53^ reached a similar approach that parameters of pressurization application are effective on %OD_620_.

**Table 4:** Experimental Results of Phenolic Acids and Anthocyanin Compounds (mg/L) of Shalgam Samples after HHP Application^a^,^b^

	responses										
process number	gallic acid	caffeic acid	P-coumaric acid^b^	ferulic acid^b^	chlorogenic acid	sinapic acid	catechin	delphinidin-3-O-glucoside^b^	cyanidin-3-O-glucoside^b^	malvidin 3-O-glucoside^b^	peonidin-3-O-glucuside^b^
C	77.99^ı^	25.74^fgh^	1.83	9.49	110.05^m^	0.86^ı^	241.86^p^	6.39	210.40	8.15	15.63
1	78.05^h^	25.70^h^	1.99	9.48	115.02^h^	0.86^ı^	366.86^n^	4.17	151.47	17.12	32.84
2	78.58^cd^	25.95^b^	2.03	9.65	118.74^f^	1.04^fg^	428.66^j^	5.57	204.64	19.78	37.94
3	78.63^c^	25.78^efg^	1.90	9.31	113.03^ı^	1.03^fgh^	364.92°	3.17	119.36	10.64	20.41
4	79.24^a^	25.74^fgh^	1.98	9.86	107.68^n^	1.26^d^	494.77^d^	4.25	157.50	14.92	28.62
5	78.27^f^	25.81^de^	1.94	9.59	110.04^m^	0.98^h^	455.35^f^	3.95	144.92	15.97	30.63
6	78.40^e^	25.91^bc^	2.04	9.81	115.51^g^	1.20^de^	483.24^e^	4.47	163.27	18.33	35.15
7	78.56^d^	25.85^cd^	1.95	9.57	120.82^e^	1.08^f^	396.78^l^	3.60	133.53	13.89	26.65
8	78.84b	25.83^de^	2.02	9.94	125.61^d^	1.34^c^	574.48^c^	5.03	182.61	18.80	36.06
9	78.37^e^	26.07^a^	2.05	9.80	162.25^b^	1.44^b^	599.14^b^	5.46	201.45	21.00	40.28
10	78.53^d^	25.73^gh^	1.93	9.77	111.50^l^	1.17^e^	384.32^m^	3.42	126.69	12.79	21.53
11	78.20^g^	26.13^a^	2.08	10.12	170.36^a^	1.54^a^	635.53^a^	5.85	216.04	23.89	45.83
12	78.63^c^	25.90^bc^	1.99	9.61	128.90^c^	1.00^gh^	422.99^k^	3.93	144.29	14.59	27.99
13	78.38^e^	25.80^def^	1.96	9.79	111.82^k^	1.18^e^	431.95^h^	3.94	145.24	16.14	27.96
14	78.37^e^	25.79^efg^	1.94	9.74	111.56^l^	1.20^de^	432.85^g^	3.83	144.20	15.89	27.68
15	78.40^e^	25.79^efg^	2.00	9.80	112.02^j^	1.16^e^	430.78^ı^	4.00	147.16	16.44	28.16

a Different small letters represent significant differences (*p* < 0.05).

b RSM was performed.

### Effect of HHP on Bioactive Compounds

Bioactive compound results of pressurized and control samples are summarized in Table 2. Chang et al.^35^ emphasized that higher phenolic contents of 25.26 and 26.81 mg/mL were determined after HHP application at 300 and 600 MPa for 3 min at 20 °C in comparison with the control (23.47 mg/mL) and also there was no important effect of pressurization on the anthocyanin content. In addition to a previous study, Torres-Ossandón et al.^23^ mentioned for white grape juice that an increase of phenolic content was observed after all application conditions of HHP (200, 300, 400 MPa; 2, 4 min; 20 °C), but this increase at levels above 300 MPa for 2 min in terms of antioxidant retention. However, in the study performed on wine, the maximum anthocyanin level was reached at the lowest pressure level (200 MPa; 20 °C; 10 min).^32^ Both TPC and TAA values in our study were not differentiated significantly (*p* > 0.05) in terms of pressurization conditions except for the term of square of time (*p* < 0.05). Compared to the control shalgam sample, it was observed that the TPC value increased under 300 MPa-30 °C-m10 min conditions, and there was a loss in TAA measurements for all HHP conditions. However, it was determined that the losses were less in the 10 min application at almost all pressure values in the TAA value.

**Figure 4:**
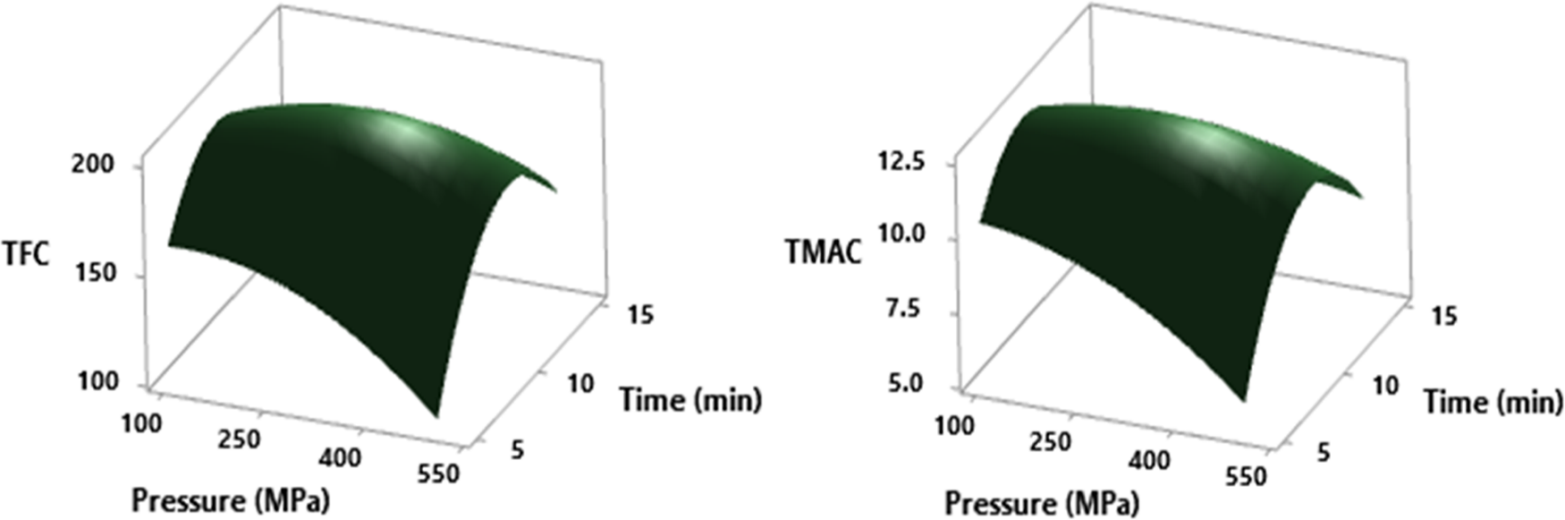
RSM plot of the HHP effect on TFC (mg QE/100 mL) and TMAC (mg/100 mL) values in cross-interaction between pressure and time parameters at a hold value (temperature of 30 °C).

In the cases of TFC and TMAC, these compounds were affected in the same way (Figure 4). When tables of analysis of variance are investigated, the only factor that causes a statistically meaningful change is observed as the square of time for these measurements (*p* = 0.014 and *p* = 0.015). TFC was 186.85 ± 23.49 mg QE/100 mL and TMAC was 13.27 ± 0.57 mg/100 mL in the control sample. Under the experimental circumstances of pressure ∼100 to 350 MPa, temperature ∼25 to 35 °C, and duration ∼7 to 12 min, the graph plot was observed at a maximum level of TFC. Additionally, pressure ∼100 to 350 MPa, temperature ∼30 to 38 °C, and duration ∼8 to 12 min in the graph plot was observed at the maximum level of TMAC. When the maximum levels were obtained for TFC, a tendency to reach higher levels than the control sample was obtained, while the maximum level for TMAC remained below the control level value. In other words, in terms of the TMAC values, it was concluded that there were losses in general. Torres-Ossandón et al.^23^ reported for grape juice concentrate that there were no significant differences between HHP-treated samples and control samples at 200, 300, and 400 MPa for 2 and 4 min at room temperature in terms of the total flavonoid content. In the study of citrus-maqui beverages, minor statistically important changes in the flavonoid levels and slightly lower levels of anthocyanin contents were observed after treatment at 450 and 600 MPa for 3 min at 20 °C.^38^ Although the degradation of anthocyanins is due to many reasons such as pH change and temperature effect, one of the most prominent among these in terms of our results was the activity of enzymes in the environment. In our experiments, we observed a decrease in the anthocyanin level in the same direction as the increasing reducing sugar concentration, which was dependent on the square of the pressurization time. It was thought that it may be an indication that anthocyanins are breaking down into anthocyanidins and sugars because of especially the increasing activities of the β-glucosidase, β-galactosidase, and α-arabinosidase enzymes with pressure application.^54^

### Effect of HHP on Phenolic Acid and Anthocyanin Profiles

Phenolic and anthocyanin profiles and their quantitation of HHP-treated shalgam samples are summarized in Table 4. As stated in many previous studies,^40,55^ one of the most dominant phenolic acid components in shalgam is caffeic, which is in line with our findings (25.70–26.07 mg/L). The most expected bioactive compound found in shalgam beverage due to the turnip plant and black carrot in its composition is catechin.

**Figure 5:**
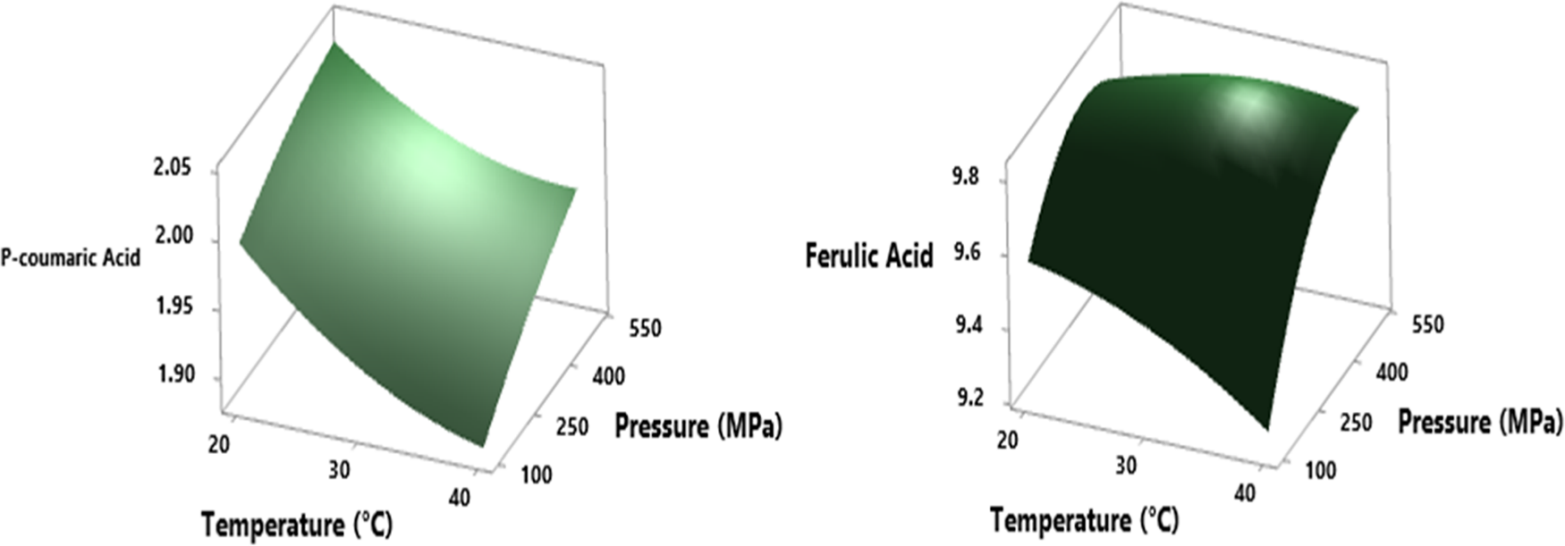
RSM plot of the HHP effect on p-coumaric and ferulic acid (mg/L) values in cross-interaction between pressure and time parameters at a hold value (time of 10 min).

All ten components, except for gallic acid, reached their highest levels after the process involving mid-pressure, the lowest temperature, and the longest pressurization time (300 MPa-20 °C-15 min) (Table 4). When the results were examined from a general perspective, a tendency toward an increase in phenolic and anthocyanin compounds was observed, with the results varying according to different conditions in the pressurization application. For RSM results (Figure 5), the p-coumaric acid content showed significant changes inversely correlated to the temperature increase and directly to the pressure increase (*p* < 0.05). Additionally, ferulic acid contents have a statistically important change proportional to the pressure and the square of the pressure (*p* < 0.05) (Figure 5). Considering the ANOVA results, a significant linear change in the amount of gallic acid with pressure and temperature was detected, while changes in the amount of caffeic and chlorogenic acid with temperature linearly and with square of the time parameter were observed.

**Figure 6:**
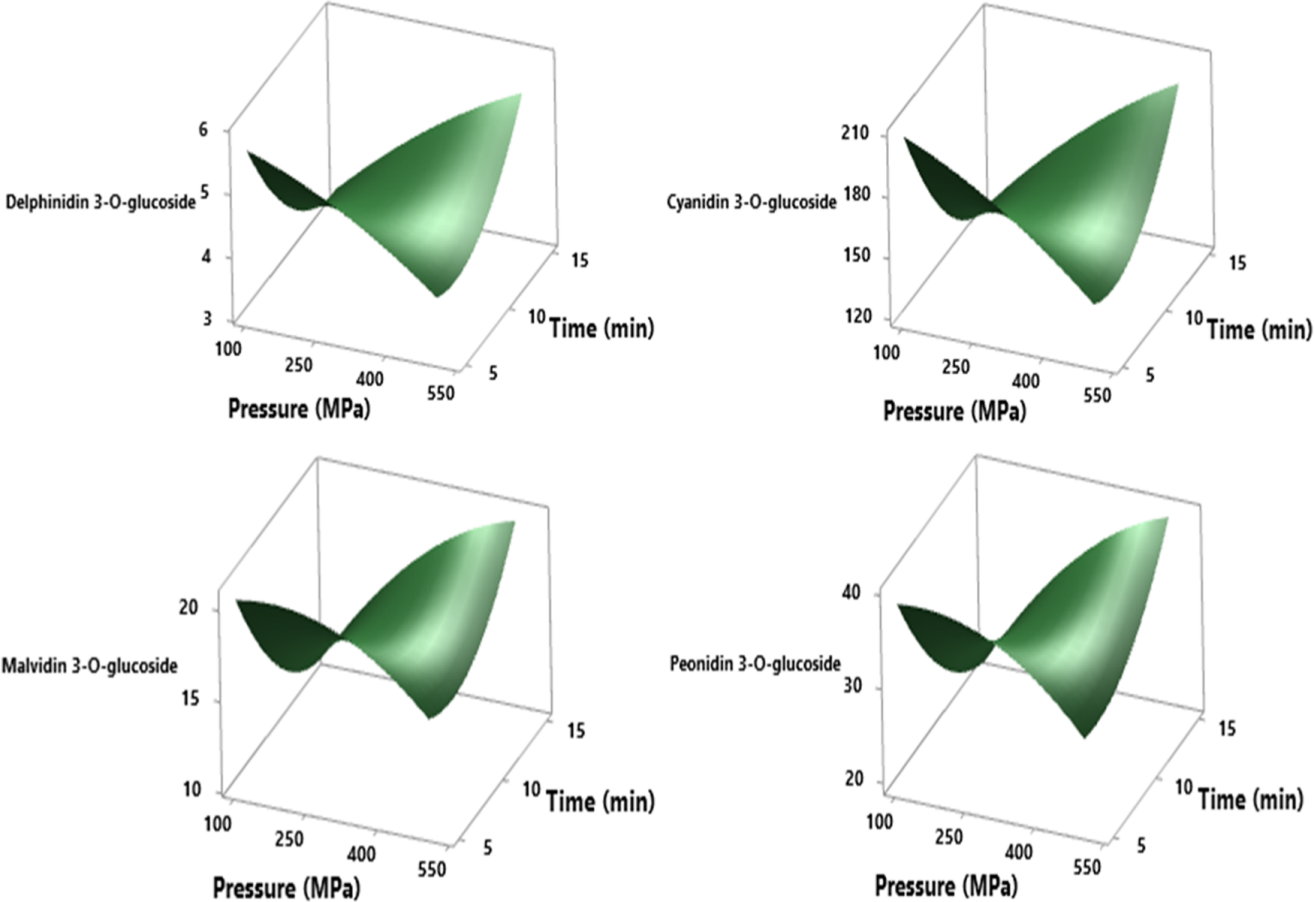
RSM plot of the HHP effect on delphinidin-3-O-glucoside, cyanidin-3-O-glucoside, malvidin 3-O-glucoside, and peonidin-3-O-glucoside (mg/L) values in cross-interaction between pressure and time parameters at a hold value (temperature of 30 °C).

Shalgam also has a very rich composition in terms of anthocyanins and contains high amounts of cyanidin and cyanidin-based sugars due to especially black carrot (cyanidin-3-O-galactoside, cyanidin-3-O-glucoside, cyanidin-3-xylosylglucosylgalactoside, cyanidin-3-xylosylgalactoside, etc.).^55,56^ Compared to other anthocyanin components, it is seen that the cyanidin-3-O-gluciside compound is dominant in all turnip juice samples, and the amount of this compound increases significantly with HHP application (Table 4). At condition 300 MPa-20 °C-15 min, delphinidin-3-O-glucoside, cyanidin-3-O-glucoside, malvidin 3-O-glucoside, and peonidin-3-O-glucoside have the highest levels as 5.85, 216.04, 23.89, and 45.83 mg/L, respectively. RSM results of all anthocyanin compounds prove that significant changes in values occur as a result of the interaction of the pressure level and application time (Figure 6).

### Model Fitting

A significant lack of fit indicates that the models were unsuccessful in representing the data within the experiment, particularly where certain points were not considered in the regression analysis.^57^ Modeling of responses (Y&M reduction, TA, RSC, TFC, TMAC, b*, C*, *h*°, BTC, p-coumaric acid, ferulic acid, delphinidin-3-O-glucoside, cyanidin-3-O-glucoside, malvidin 3-O-glucoside, and peonidin-3-O-glucoside) were successfully performed and are summarized in Table 5. Also, the coefficients of the fitted second-order polynomial model equations are shown for the nine responses, indicating insignificant lack-of-fit values, except for phenolic and anthocyanin compounds, in Table 6. Although the determination coefficient values were obtained as high, lack-of-fit values are not in compliance with some results in the model (TMAB, LAB, pH, TSS, a*, IC, gallic, caffeic, chlorogenic, sinapic acids, and catechin).

**Table 5:** *P*-Values in Analysis of Variance (ANOVA) of the RSM Second-Order Polynomial Model^a^

source	Y&M	TA	RSC	TFC	TMAC	b*	C*	*h*°	BCT	P-coumaric Acid	ferulic acid	delphinidin-3-O-glucoside	cyanidin-3-O-glucoside	malvidin 3-O-glucoside	peonidin-3-O-glucoside
model	0.000	0.014	0.004	0.152	0.136	0.311	0.278	0.344	0.044	0.055	0.032	0.415	0.374	0.325	0.489
linear	0.000	0.012	0.002	0.653	0.335	0.323	0.263	0.336	0.486	0.011	0.018	0.682	0.685	0.404	0.577
X1	0.000	0.003	0.001	0.294	0.264	0.276	0.61	0.218	0.637	0.016	0.005	0.709	0.745	0.259	0.352
X2	0.000	0.236	0.031	0.605	0.173	0.178	0.153	0.202	0.307	0.006	0.124	0.454	0.451	0.541	0.524
X3	0.001	0.276	0.018	0.816	0.652	0.493	0.191	0.694	0.308	0.328	0.372	0.419	0.417	0.275	0.452
square	0.000	0.016	0.004	0.043	0.045	0.148	0.204	0.159	0.014	0.241	0.058	0.471	0.420	0.363	0.574
X1 * X1	0.000	0.008	0.035	0.255	0.372	0.89	0.811	0.798	0.032	0.666	0.030	0.650	0.668	0.314	0.392
X2 * X2	0.048	0.046	0.002	0.094	0.083	0.889	0.731	0.966	0.017	0.279	0.368	0.858	0.871	0.975	0.874
X3 * X3	0.087	0.046	0.007	0.014	0.015	0.035	0.05	0.039	0.037	0.095	0.099	0.174	0.145	0.178	0.337
2-way interaction	0.004	0.048	0.286	0.348	0.416	0.668	0.388	0.776	0.083	0.776	0.100	0.193	0.172	0.211	0.263
X1 * X2	0.005	0.472	0.782	0.961	0.579	0.406	0.304	0.46	0.02	0.492	0.102	0.872	0.815	0.855	0.950
X1 * X3	0.006	0.012	0.165	0.182	0.166	0.986	0.649	0.854	0.746	0.578	0.464	0.049	0.045	0.062	0.078
X2 * X3	0.017	0.385	0.19	0.241	0.529	0.402	0.201	0.535	0.431	0.659	0.055	0.657	0.563	0.436	0.510
lack-of-fit	0.659	0.077	0.09	0.059	0.09	0.766	0.366	0.858	0.764	0.615	0.067	0.099	0.100	0.249	0.170
*R*^2^	0.99	0.94	0.96	0.82	0.83	0.74	0.76	0.73	0.90	0.89	0.91	0.70	0.71	0.74	0.66
*R*^2^(adj)	0.99	0.83	0.90	0.51	0.53	0.28	0.33	0.24	0.72	0.70	0.76	0.15	0.20	0.26	0.05

a X1, pressure level (MPa); X2, application temperature; X3, application time.

**Table 6:** Estimated Regression Coefficient (coded) of the RSM Second-Order Polynomial Model^a^,^b^

	responses								
coefficients	Y&M reduction	TA	RSC	TFC	TMAC	b*	C*	*h*°	BCT
β_0_ (constant)	3.8167	2.493	7.209	198.0	12.19	23.674	59.207	0.4113	4.5309
Linear									
β_1_	1.345	–0.45	–2.497	–10.08	–0.795	–0.542	–0.159	–0.00899	0.0192
β_2_	0.5275	–0.11	1.017	4.74	1.005	0.695	0.491	0.00938	–0.0434
β_3_	0.3825	–0.1	–1.176	2.11	–0.303	–0.328	–0.442	–0.00266	–0.0434
Square									
β_11_	–1.1446	0.518	1.439	–16.3	–0.913	–0.095	0.108	–0.00254	0.1652
β_22_	–0.1946	0.318	2.855	–26.1	–2.015	0.096	0.156	0.00042	0.1987
β_33_	–0.1596	0.318	2.197	–46.6	–3.405	1.877	1.107	0.02612	–0.1594
Interaction									
β_12_	0.3475	0.09	–0.141	0.6	–0.531	0.57	0.473	0.00722	–0.1822
β_13_	0.3275	0.45	0.783	18.8	1.45	–0.012	0.2	–0.00175	0.0185
β_23_	0.2525	0.11	0.731	–16.1	–0.605	0.574	0.609	0.006	–0.0463

a *Y* = β_0_ + β_1_*X*_1_ + β_2_*X*_2_ + β_3_*X*_3_ + β_11_*X*_1_^2^ + β_22_*X*_2_^2^ + β_33_*X*_3_^2^ + β_12_*X*_1_*X*_2_ + β_13_*X*_1_*X*_3_ + β_23_*X*_2_*X*_3_ + ε.

b *X*_1_, pressure level (MPa); *X*_2_, application temperature; *X*_3_, application time.

### Model Optimization and Verification

For the purpose of obtaining the highest possible values for Y&M reduction, TFC, and TMAC and the lowest values for TA and RSC, the optimization method was used to predict the ideal level of independent variables. Color values (b*, C*, *h*°, and BCT) were not included in model optimization due to better desirability, but the results obtained as a result of optimum conditions were checked for compliance with the targeted values. It was observed that the desired value (0.93) of the modeling optimization performed in this way was much higher than when the color parameters were included (0.74), and the color values were obtained closer to the target values. The optimum condition parameters of HHP application were a pressure level of 367 MPa, process temperature of 31.9 °C, and process time of 10.5 min to obtain the desired quality of shalgam. This ideal condition produced the values of Y&M reduction (4.30 log cfu/mL), TFC (192.89 mg QE/100 mL), TMAC (11.88 mg/100 mL), TA (2.41 g_lactic acid_/L), and RSC (6.78 mg/100 mL). No important differences were observed between the predicted values and the mean of experimental results for Y&M reduction (4.45 ± 0.04 log cfu/mL), TFC (194.71 ± 1.08 mg QE/100 mL), TMAC (12.05 ± 0.14 mg/100 mL), TA (2.51 ± 0.21 g_lactic acid_/L), RSC (6.68 ± 0.07 mg/100 mL), b* (24.86 ± 1.53), C* (57.94 ± 0.33), *h*° (0.43 ± 0.03), BCT (4.75 ± 0.18%), p-coumaric acid (2.04 mg/L), ferulic acid (9.83 mg/L), delphinidin-3-O-galactoside (4.06 mg/L), cyanidin-3-O-glucoside (153.76 mg/L), malvidin-3-O-glucoside (16.98 mg/L), and peonidin-3-O-glucoside (29.45 mg/L).

## Conclusions

High-pressure processing (HHP) involves applying extremely high pressure to food to kill or inactivate microorganisms and enzymes, prolonging the product’s shelf life while preserving its nutritional and sensory attributes. Although HHP itself does not directly generate green energy, it can play a role in reducing energy consumption, food waste, and the environmental impact of the food industry. By encouraging effective and eco-friendly food preservation techniques, it supports sustainability objectives. This research concluded that HHP is a promising nonthermal food preservation technology for shalgam, with the potential to enhance its visual appeal and retain its freshness qualities while ensuring microbial inactivation. The optimum HHP conditions were determined in terms of pressure level, temperature, and time parameters as 367 MPa, 31.9 °C, and 10.5 min, respectively. For this condition, the values of Y&M reduction, TFC, TMAC, TA, and RSC values were obtained as 4.30 log cfu/mL, 192.89 mg QE/100 mL, 11.88 mg/100 mL, 2.41 g_lactic acid_/L, and 6.78 mg/100 mL, respectively. The other microbiological reduction values for TMAB and LAB at the optimum condition were <5.00 and 6.68 (total inactivation) log cfu/mL, respectively. Sensory factors such as appearance, color, and odor are very important criteria for shalgam consumption, and it has been observed that HHP application does not have a negative effect on these quality characteristics. In particular, the results obtained for the basic color parameters proved that there was no significant change in the saturated red color of the shalgam (a* and RCT). For phenolic and anthocyanin components, caffeic acid, catechin, and cyanidin-3-O-glucoside derivatives are the most frequently found in turnip plants. Gallic acid, caffeic acid, chlorogenic acid, catechin, cyanidin-3-O-glucoside, malvidin-3-O-glucoside, and peonidin-3-O-glucoside were detected as dominant compounds in shalgam samples, and their concentrations increased with HHP application. Furthermore, it was noted that despite variations in the temperature and pressure based on the HHP parameters, the bioactive components did not exhibit a discernible increase or decrease. Additionally, innovations in the application of HHP may lead to further synergies with green energy, such as the development of more energy-efficient HHP equipment or the integration of renewable energy sources into HHP processes.
